# Neighborhood Deprivation and Risk of Cancer Incidence, Mortality and Survival: Results from a Population-Based Cohort Study in Japan

**DOI:** 10.1371/journal.pone.0106729

**Published:** 2014-09-03

**Authors:** Yasuhiro Miki, Manami Inoue, Ai Ikeda, Norie Sawada, Tomoki Nakaya, Taichi Shimazu, Motoki Iwasaki, Taiki Yamaji, Shizuka Sasazuki, Kenji Shibuya, Shoichiro Tsugane, for the JPHC Study Group

**Affiliations:** 1 Graduate School of Medicine, The University of Tokyo, Tokyo, Japan; 2 Research Center for Cancer Prevention and Screening, National Cancer Center, Tokyo Japan; 3 Department of Geography, Ritsumeikan University, Kyoto, Japan; Universität Bochum, Germany

## Abstract

**Background:**

In many developed countries, socioeconomic status is associated with cancer incidence and survival. However, research in Japan is sparse. We examined the association between neighborhood deprivation based on the Japanese Deprivation Index and the risk of incidence, mortality and survival from total and major cancers in the Japan Public Health Center-based Prospective Study.

**Methods:**

86,112 participants were followed through the end of 2009. A total of 10,416 incident cases and 5,510 deaths from cancer were identified among 1,348,437 person-years of follow-up (mean follow-up: 15.7 years). The Japanese deprivation index was used to access neighborhood deprivation. Hazard ratios and 95% confidence intervals were calculated by Cox regression analysis.

**Results:**

We found no associations between neighborhood deprivation index and the incidence of total and major cancers. In some cancer risks or deaths, however, we found positive or inverse associations with a higher deprivation index, such as a decreased risk of colorectal cancer incidence and an increased risk of liver cancer incidence and deaths in women.

**Conclusion:**

Although some positive or inverse associations were detected for specific sites, the neighborhood deprivation index has no substantial overall association with the risk of incidence, mortality and survival from cancer in the Japanese population.

## Introduction

In many developed countries, socioeconomic status (SES) is associated with cancer incidence and survival [1]–[6]. Although this association is not completely understood, some cancers are thought to be caused by lifestyle habits in the community, such as smoking, which tend to be concentrated among the poorer segments of a population [7]. Possible explanations for the association between SES and cancer survival include a delay in diagnosis and poor access to treatment in low SES groups [8]. Although Japan has an extensive welfare system and equal access to health care [9], one study showed associations between poor gastric cancer survival and job status, such as unemployment and manual labor [10].

Although socioeconomic position is usually expressed in terms of individual income, education, and/or occupation, areal deprivation, as a group factor, is also used to express relative poverty in area [11]. One Japanese study in a metropolitan area showed an inverse association between cervical and endometrial cancer survival and areal deprivation according to area unemployment rate [12]. However, no population-based study with a wider focus on the association between areal deprivation and cancer has yet appeared.

Here, we evaluated the association between the incidence, mortality and survival of all sites and major sites of cancer in a large population-based cohort study and the Japanese Deprivation Index (JDI), an index of neighborhood deprivation suitable for use in Japan. We wanted to test if areal deprivation increases the risk of incidence, mortality and survival of all cancers and major cancer sites in a non-metropolitan cohort setting.

## Materials and Methods

### Ethics Statement

The protocol of the Japan Public Health Center-based Prospective Study (JPHC Study) was approved by the Institutional Review Board of the National Cancer Center, Japan (approval number: 13-021). We informed the detail of the study to all study subjects orally and/or in writing at baseline survey. In our study we did not get written informed consent from all participants since our study was initiated in 1990, which was before ethical guideline for epidemiologic studies was enforced in 2002 in Japan. Instead, we informed the detail of the study to all study participants not only at baseline survey, but also through mails several times during follow-up. The protocol of the study including this was approved by the institutional review board annually since 2001, based on current ethical guideline.

### Study Populations

Study participants were Japanese inhabitants enrolled in the JPHC Study Cohorts I and II, a large-scale population-based study. Details of the JPHC study have been provided elsewhere [13], [14]. Briefly, the study was launched in January 1990 (Cohort I) and 1993 (Cohort II), and covered five and six public health center (PHC)-based areas, respectively.

In the baseline study, we identified 117,125 men and women aged 40–59 years for Cohort I and 40–69 years for Cohort II, with two areas excluded because information on cancer incidence and/or deprivation index was not available. During the follow-up period, 229 participants were excluded because of erroneous identification (n = 170), non-Japanese nationality (n = 51), duplicate registration (n = 4) and unsuitable age (n = 4). 95,292 participants responded to the questionnaires (81.5% response rate). At this point, participants with a history of cancer at baseline (n = 2,038) and those with insufficient information related to deprivation and other confounders (n = 7,142) were excluded. Finally, we identified a study population of 86,112 men and women (40,883 men and 45,229 women).

### Assessment of deprivation

Neighborhood deprivation was assessed using the JDI, a deprivation index developed by Nakaya [15], [16]. The JDI is a composite indicator which consists of weighted sums of a number of census-based variables calculated using the same method as the Breadline Britain poverty measure [17] and European transnational ecological deprivation measure [18], [19]. Deprivation-related variables were obtained from the 1995 population census.

### Follow-up

We followed study participants from the baseline survey until December 31, 2009. Changes in residence status, including survival, were confirmed annually by the residential registry. Among study participants, 14,075 died, 6,911 moved out of the study area, 6 withdrew from the study, and 305 (0.3 percent) were lost to follow-up within the follow-up period. Due to a lack of precise cancer incidence data outside the study area, those who moved out of the study area were censored at the time they moved. For those who moved within the study area, we did not consider changing neighborhood deprivation status.

### Confirmation of cancer mortality

Information on the cause of death for deceased participants was obtained from death certificates (provided by the Ministry of Health, Labour, and Welfare, with permission), which include cause of death as defined according to the International Classification of Diseases, Tenth Revision [20]. Resident registration and death registration are required by law in Japan, and the registries are believed to be complete. During the follow-up period, 5,510 cancer deaths were identified.

### Confirmation of cancer incidence

The occurrence of cancer was identified by active patient notification from major local hospitals in the study area and from data linkage with population-based cancer registries, with permission from each of the local governments responsible for the cancer registries. When incidence data were unavailable, death certificate information was used as a supplementary information source. Cancer sites included in this study were coded according to the International Classification of Diseases for Oncology, Third Edition (ICD-O-3) [21]. In our cancer registry system, the proportion of cancer cases ascertained by death certificate only (DCO) was 5.6%. For the present analysis, the earliest date of diagnosis was used in cases with multiple primary cancers diagnosed at different times. We identified 10,416 newly diagnosed cancer cases during the follow-up period.

### Statistical analysis

We prospectively counted the number of person-years of follow-up for each subject from the date of completion of the questionnaire until the date of diagnosis of cancer, date of death, movement out of the study area, or end of the study period (December 31, 2009), whichever occurred first. For cancer cases, person-years were further calculated for survival analysis from the date of cancer diagnosis until December 31, 2011.

A Cox proportional hazards regression model with the clustered sandwich estimator was applied to calculate the multivariate-adjusted hazard ratio (HR) and 95% confidence interval (CI) of associations between quartile of neighborhood deprivation index and incidence, mortality, and survival of all sites of cancer and cancer at major sites, namely stomach (ICD-O-3: C16), colon (C18), rectum (C19, C20), liver (C22), pancreas (C25), lung (C34), breast (C50) and prostate (C61). STATA version 12.0 (Stata Corp, College Station, TX) [22] was used for all analyses.

We used the multivariate model including age (continuous) (age at diagnosis for cancer survival) and study area (nine public health centers), population density (quartile), occupation (professional or office worker, sales clerk or other, farmer, or manual laborer and unemployed), smoking (never, past, current), alcohol drinking (none, ≤150 ml, 150–300 ml, 300–450 ml, ≥450 ml for men and none, ≤150 ml, >150 for women), body mass index (BMI) (≤18, ≥30), and leisure time sport activity (none, once per 1–3 months, more than once a week). These variables, obtained from the questionnaire, are either known or suspected from earlier studies to be risk factors for outcome. For incidence and survival of prostate cancer, HRs were also estimated by clinical stage (i.e., localized or advanced). We also implemented additional sensitivity analyses which included education as a confounder (junior high school, high school and college or higher) to the multivariate model among Cohort I only (n  = 38,340) due to a lack of education information among Cohort II.

The proportional hazards assumptions were tested using scaled Schoenfeld residuals and a graphical plot of the cumulative rate on a log scale, and no violation was found. We calculated *p* values for the analysis of linear trends using the median values of each neighborhood deprivation index category in the regression model. All reported *p* values are two-tailed.

## Results

Tables 1 and 2 compares the characteristics of participants according to neighborhood deprivation index quartile. For men (Table 1), those with a higher neighborhood deprivation index were more likely to be older, a farmer or unemployed, a non-smoker, and more obese, and less likely to have undergone cancer screening. Similar trends were observed for women (Table 2). Women with a high neighborhood deprivation index tended to drink less alcohol, but showed no difference in drinking behavior pattern to those with a low index. No consistent trends in the proportion of population density or frequency of leisure time sports activity were observed across neighborhood deprivation categories. Those who were lost to follow-up (n = 305, 0.3% of the study participants) tended to be younger than those who were not lost, but had substantially similar baseline characteristics for other variables.

**Table 1:** Baseline characteristics in men (n = 40,883).

	Deprivation Index Quartile			
	Lowest	Second	Third	Highest
Median	478.8	530.8	591.3	698.3
[Min-Max]	[165.8–504.2]	[504.7–555.8]	[556.5–624.4]	[627.6–983.3]
Number of participants	10,532	10,713	9,766	9,872
Age (%)				
≤44	25	25.2	23.9	23
45–49	20.7	20.9	20.1	17.5
50–54	19.2	19.9	20.5	19.2
≥55	35.1	34	35.5	40.3
Population density (%)				
Lowest	11	21.5	31.5	34.8
Second	36.6	25.8	19.3	20.4
Third	18.2	28.6	39.8	13.8
Highest	34.2	24.1	9.4	31.1
Occupation (%)				
Professionals and office workers	34.7	29.1	24	19.1
Sales clerk or others	14.9	16.8	16.9	15.6
Farmers	17.3	17.8	27.3	32.5
Manual laborers	27.6	31.5	27.1	26.6
Unemployed	5.5	4.8	4.7	6.3
Smoking (%)				
Never smoking	19.1	22.4	25.5	30.3
Past smoking	24	22.9	21.4	23.8
Current smoking	56.9	54.7	53.1	46
Weekly alcohol consumption				
None	20.9	19.7	22.1	27
≤150 ml	32	34.2	34.6	36.2
150–300 ml	25.1	23.8	21.4	15.8
300–450 ml	13.5	14.2	13.3	11.1
≥450 ml	7.5	8.1	8.6	9.9
BMI (%)				
≤18	4.4	4.7	3.6	3.6
≥30	1.5	1.8	2.2	3.2
Sports (%)				
None	65.3	63.6	66.3	69.4
Once per 1–3 months	16.8	17.5	15.3	11.5
More than once a week	17.9	18.9	18.4	19.2
History of cancer screening (%)				
No	17.2	18.7	20.5	22.3
Yes	82.8	81.3	79.5	77.7

**Table 2:** Baseline characteristics in women (n = 45,229).

	Deprivation Index Quartile			
	Lowest	Second	Third	Highest
Median	478.8	531.6	591.3	698.3
[Min-Max]	[165.8–504.2]	[504.7–555.8]	[556.5–624.4]	[627.6–983.3]
Number of participants	11,121	11,686	11,126	11,296
Age (%)				
≤44	23.5	23.1	22.6	19.8
45–49	19.7	20.3	19.6	16.8
50–54	18.7	20.7	21.5	19.9
≥55	38.1	35.9	36.4	43.6
Population density (%)				
Lowest	11.2	20.9	29.5	36.7
Second	36.8	25.2	20.4	21.9
Third	17.5	28.6	41.3	12.1
Highest	34.5	25.3	8.9	29.4
Occupation (%)				
Professionals and office workers	18.2	16.7	15.1	12.2
Sales clerk or others	13.4	16.4	15.4	14.4
Farmers	18.9	18.8	27.3	29.4
Manual laborers	16	18	13.8	8.4
Unemployed	33.6	30.1	28.4	35.6
Smoking (%)				
Never smoking	92.5	92.4	93	93.4
Past smoking	1.3	1.2	1.6	1.4
Current smoking	6.2	6.4	5.5	5.2
Weekly alcohol consumption				
None	74.8	76.2	79	86.8
≤150 ml	11.3	11.1	10.3	7
>150 ml	13.9	12.7	10.7	6.3
BMI (%)				
≤18	5.8	5.8	5	4.6
≥30	2.4	2.6	3.6	4.5
Sports (%)				
None	76.2	75.1	75.9	77.3
Once per 1–3 months	7.2	7.6	7	6
More than once a week	16.6	17.3	17.2	16.8
History of cancer screening (%)				
No	17.2	17.1	17.7	20.9
Yes	82.8	82.9	82.3	79.1

Tables 3 and 4 showed that neighborhood deprivation index was generally not associated with the risk of total cancer incidence, mortality or survival in either men or women, although we observed some significant inverse or positive associations in specific sites of cancer.

**Table 3:** Deprivation index and cancer risk in men (n = 40,883).

Deprivation Index Quartile		Number of participants	Number of cases	Number of deaths	Number of deaths among cases	Incidence			Death			Survival		
						HR*	(95%CI)		HR*	(95%CI)		HR*	(95%CI)	
All sites			6,360	3,013	3,678									
	Lowest	10,532	1,693	827	985	1.00			1.00			1.00		
	Second	10,713	1,650	749	919	0.94	(0.87–	1.01)	0.94	(0.84–	1.04)	1.01	(0.95–	1.07)
	Third	9,766	1,453	679	840	0.96	(0.88–	1.06)	0.94	(0.83–	1.07)	1.01	(0.95–	1.09)
	Highest	9,872	1,564	758	934	0.90	(0.80–	1.00)	0.87	(0.74–	1.01)	1.00	(0.92–	1.09)
	*p for trend*					*0.086*			*0.083*			*0.968*		
*Stomach (C16)*			1,383	482	712									
	Lowest	10,532	444	173	244	1.00			1.00			1.00		
	Second	10,713	393	125	187	0.95	(0.82–	1.10)	0.83	(0.64–	1.06)	0.93	(0.81–	1.08)
	Third	9,766	294	102	149	0.95	(0.79–	1.15)	0.90	(0.64–	1.26)	0.95	(0.79–	1.15)
	Highest	9,872	252	82	132	0.95	(0.76–	1.19)	0.80	(0.53–	1.20)	0.91	(0.73–	1.14)
	*p for trend*					*0.679*			*0.329*			*0.471*		
*Colorectum (C18-C20)*			1,207	307	474									
	Lowest	10,532	320	89	127	1.00			1.00			1.00		
	Second	10,713	315	72	121	0.91	(0.75–	1.09)	0.89	(0.66–	1.21)	1.08	(0.88–	1.34)
	Third	9,766	287	62	105	0.93	(0.74–	1.16)	0.92	(0.60–	1.41)	1.07	(0.83–	1.36)
	Highest	9,872	285	84	121	0.75	(0.57–	0.98)	1.02	(0.63–	1.67)	1.11	(0.82–	1.50)
	*p for trend*					*0.050*			*0.852*			*0.592*		
*Colon (C18)*			800	172	278									
	Lowest	10,532	203	40	69	1.00			1.00			1.00		
	Second	10,713	208	47	75	0.97	(0.78–	1.21)	1.30	(0.83–	2.05)	1.07	(0.81–	1.42)
	Third	9,766	186	30	55	1.04	(0.79–	1.36)	1.01	(0.53–	1.92)	0.87	(0.61–	1.24)
	Highest	9,872	203	55	79	0.87	(0.62–	1.24)	1.21	(0.59–	2.50)	1.01	(0.68–	1.51)
	*p for trend*					*0.467*			*0.707*			*0.966*		
*Rectum (C19*–*C20)*			407	135	196									
	Lowest	10,532	117	49	58	1.00			1.00			1.00		
	Second	10,713	107	25	46	0.79	(0.60–	1.04)	0.56	(0.34–	0.92)	1.11	(0.80–	1.53)
	Third	9,766	101	32	50	0.75	(0.52–	1.06)	0.84	(0.45–	1.55)	1.40	(1.04–	1.88)
	Highest	9,872	82	29	42	0.54	(0.36–	0.82)	0.85	(0.41–	1.79)	1.27	(0.81–	1.98)
	*p for trend*					*0.007*			*0.871*			*0.185*		
*Liver (C22)*			425	366	378									
	Lowest	10,532	104	94	86	1.00			1.00			1.00		
	Second	10,713	112	94	99	1.20	(0.92–	1.57)	1.10	(0.84–	1.46)	1.06	(0.96–	1.16)
	Third	9,766	89	78	87	1.16	(0.82–	1.63)	1.07	(0.75–	1.53)	1.15	(1.03–	1.28)
	Highest	9,872	120	100	106	1.23	(0.82–	1.85)	0.91	(0.59–	1.40)	1.03	(0.89–	1.20)
	*p for trend*					*0.426*			*0.545*			*0.681*		
*Pancreas (C25)*			188	183	176									
	Lowest	10,532	54	52	51	1.00			1.00			1.00		
	Second	10,713	45	48	44	0.85	(0.55–	1.31)	0.92	(0.58–	1.46)	1.05	(0.95–	1.15)
	Third	9,766	50	48	48	1.13	(0.71–	1.82)	1.13	(0.68–	1.87)	1.04	(0.95–	1.15)
	Highest	9,872	39	35	33	0.99	(0.52–	1.87)	1.04	(0.55–	1.96)	0.92	(0.77–	1.11)
	*p for trend*					*0.857*			*0.784*			*0.352*		
*Lung (C34)*			887	699	749									
	Lowest	10,532	232	183	193	1.00			1.00			1.00		
	Second	10,713	236	184	203	0.99	(0.80–	1.22)	1.03	(0.80–	1.31)	1.10	(1.01–	1.19)
	Third	9,766	193	155	167	0.84	(0.65–	1.08)	0.91	(0.68–	1.21)	1.11	(1.00–	1.23)
	Highest	9,872	226	177	186	0.88	(0.66–	1.17)	0.87	(0.62–	1.23)	1.07	(0.94–	1.21)
	*p for trend*					*0.321*			*0.344*			*0.532*		
*Prostate (C61)*			732	104	211									
	Lowest	10,532	164	20	46	1.00			1.00			1.00		
	Second	10,713	169	27	45	0.96	(0.75–	1.23)	1.24	(0.63–	2.43)	0.90	(0.62–	1.31)
	Third	9,766	219	27	64	1.56	(1.18–	2.07)	1.09	(0.52–	2.27)	0.93	(0.63–	1.36)
	Highest	9,872	180	30	56	1.04	(0.75–	1.44)	0.77	(0.35–	1.71)	0.82	(0.50–	1.34)
	*p for trend*					*0.598*			*0.254*			*0.457*		
	*Localized*		408		61									
	Lowest	10,532	108		16	1.00						1.00		
	Second	10,713	91		15	0.90	(0.66–	1.22)				1.11	(0.49–	2.52)
	Third	9,766	117		14	1.60	(1.11–	2.31)				0.61	(0.23–	1.59)
	Highest	9,872	92		16	1.16	(0.75–	1.80)				0.45	(0.14–	1.39)
	*p for trend*					*0.291*						*0.113*		
	*Advanced*		326		150									
	Lowest	10,532	57		30	1.00						1.00		
	Second	10,713	79		30	1.05	(0.72–	1.53)				0.83	(0.63–	1.35)
	Third	9,766	102		50	1.52	(1.01–	2.27)				1.20	(0.76–	1.87)
	Highest	9,872	88		40	0.95	(0.58–	1.54)				1.43	(0.78–	2.62)
	*p for trend*					*0.679*						*0.148*		

*Adjusted for age (age at diagnosis for cancer survival), area, population density (quartile), occupation (professionals and office worker, sales clerks or others, farmers, manual labors and others), smoking, alcohol drinking, body mass index, and leisure-time sport activity.

**Table 4:** Deprivation index and cancer risk in women (n = 45,229).

Deprivation Index Quartile		Number of participants	Number of cases	Number of deaths	Number of deaths among cases	Incidence			Death			Survival		
						HR*	(95%CI)		HR*	(95%CI)		HR*	(95%CI)	
*All sites*			4,056	1,603	1,832									
	Lowest	11,121	970	403	453	1.00			1.00			1.00		
	Second	11,686	1,061	415	465	1.00	(0.92–	1.10)	1.01	(0.88–	1.16)	0.99	(0.90–	1.09)
	Third	11,126	961	377	432	0.98	(0.88–	1.09)	0.96	(0.81–	1.14)	0.96	(0.85–	1.08)
	Highest	11,296	1,064	408	482	0.98	(0.86–	1.12)	0.99	(0.81–	1.22)	1.01	(0.87–	1.17)
	*p for trend*					*0.760*			*0.886*			*0.861*		
*Stomach (C16)*			576	199	234									
	Lowest	11,121	176	54	70	1.00			1.00			1.00		
	Second	11,686	162	57	60	1.00	(0.79–	1.26)	1.32	(0.92–	1.88)	1.09	(0.83–	1.43)
	Third	11,126	116	47	51	0.97	(0.72–	1.31)	1.53	(0.96–	2.43)	1.37	(0.96–	1.95)
	Highest	11,296	122	41	53	1.17	(0.81–	1.69)	1.70	(0.94–	3.09)	1.43	(0.90–	2.25)
	*p for trend*					*0.411*			*0.069*			*0.124*		
*Colorectum (C18*–*C20)*			777	213	265									
	Lowest	11,121	195	59	70	1.00			1.00			1.00		
	Second	11,686	209	52	65	1.01	(0.83–	1.22)	0.69	(0.43–	1.13)	0.80	(0.57–	1.11)
	Third	11,126	169	47	58	0.85	(0.66–	1.10)	0.51	(0.29–	0.89)	0.71	(0.48–	1.04)
	Highest	11,296	*204*	55	72	0.85	(0.63–	1.15)	0.49	(0.26–	0.90)	0.75	(0.48–	1.16)
	*p for trend*					*0.233*			*0.029*			*0.244*		
*Colon (C18)*			536	134	167									
	Lowest	11,121	138	37	45	1.00			1.00			1.00		
	Second	11,686	142	35	42	1.03	(0.80–	1.32)	0.86	(0.48–	1.53)	0.92	(0.63–	1.33)
	Third	11,126	108	24	31	0.91	(0.65–	1.25)	0.53	(0.25–	1.12)	0.70	(0.44–	1.11)
	Highest	11,296	148	38	49	1.03	(0.72–	1.47)	0.65	(0.31–	1.37)	0.78	(0.48–	1.27)
	*p for trend*					*0.927*			*0.274*			*0.277*		
*Rectum (C19*–*C20)*			241	79	98									
	Lowest	11,121	57	22	25	1.00			1.00			1.00		
	Second	11,686	67	17	23	0.93	(0.61–	1.42)	0.46	(0.21–	0.99)	0.47	(0.24–	0.92)
	Third	11,126	61	23	27	0.73	(0.46–	1.18)	0.44	(0.19–	1.02)	0.49	(0.23–	1.05)
	Highest	11,296	56	17	23	0.54	(0.32–	0.92)	0.28	(0.10–	0.76)	0.45	(0.19–	1.07)
	*p for trend*					*0.010*			*0.029*			*0.287*		
*Liver (C22)*			189	139	158									
	Lowest	11,121	38	31	36	1.00			1.00			1.00		
	Second	11,686	50	38	41	1.48	(0.93–	2.37)	1.51	(0.89–	2.54)	0.89	(0.75–	1.06)
	Third	11,126	51	36	41	1.89	(1.13–	3.15)	1.95	(1.06–	3.56)	0.89	(0.71–	1.11)
	Highest	11,296	50	34	40	1.78	(0.94–	3.36)	1.90	(0.97–	3.72)	0.90	(0.64–	1.27)
	*p for trend*					*0.105*			*0.072*			*0.344*		
*Pancreas (C25)*			172	163	164									
	Lowest	11,121	46	44	42	1.00			1.00			1.00		
	Second	11,686	42	42	41	0.75	(0.46–	1.22)	0.74	(0.45–	1.23)	1.02	(0.94–	1.11)
	Third	11,126	45	41	44	0.77	(0.43–	1.36)	0.67	(0.38–	1.17)	1.02	(0.89–	1.17)
	Highest	11,296	39	36	37	0.81	(0.43–	1.52)	0.76	(0.40–	1.46)	1.03	(0.86–	1.22)
	*p for trend*					*0.672*			*0.524*			*0.818*		
*Lung (C34)*			351	196	206									
	Lowest	11,121	79	40	43	1.00			1.00			1.00		
	Second	11,686	79	45	51	0.74	(0.53–	1.03)	0.99	(0.65–	1.52)	1.37	(1.01–	1.86)
	Third	11,126	87	48	50	0.80	(0.54–	1.19)	0.99	(0.58–	1.69)	1.11	(0.79–	1.56)
	Highest	11,296	106	63	62	0.76	(0.48–	1.21)	1.04	(0.55–	1.95)	1.21	(0.83–	1.77)
	*p for trend*					*0.467*			*0.876*			*0.732*		
*Breast (C50)*			649	105	143									
	Lowest	11,121	165	30	38	1.00			1.00			1.00		
	Second	11,686	172	27	34	1.03	(0.80–	1.31)	0.98	(0.57–	1.68)	0.97	(0.64–	1.47)
	Third	11,126	161	28	39	1.09	(0.83–	1.42)	1.08	(0.47–	2.49)	1.02	(0.59–	1.75)
	Highest	11,296	151	20	32	0.90	(0.62–	1.31)	0.66	(0.24–	1.80)	0.87	(0.46–	1.65)
	*p for trend*					*0.610*			*0.456*			*0.742*		

*Adjusted for age (age at diagnosis for cancer survival), area, population density (quartile), occupation (professionals and office worker, sales clerks or others, farmers, manual labors and others), smoking, alcohol drinking, body mass index, and leisure-time sport activity.

In men (Table 3), an inverse association was observed in colorectal cancer, particularly rectal cancer, in which men in the highest deprivation index category had decreased risks of colorectal (HR, 0.75; 95%CI, 0.57–0.98, p for trend 0.050) and rectal cancer (HR, 0.54; 95%CI, 0.36–0.82, p for trend 0.007). We also observed a sporadic positive association in prostate cancer incidence and rectal cancer mortality, albeit that no significant linear trend of risk increase by increased deprivation index was observed. No remarkable association with neighborhood deprivation index was observed for cancer survival in men.

In women (Table 4), we observed a decreased risk of incidence and mortality in rectal cancer (incidence: HR for the highest quartile, 0.54; 95%CI, 0.32–0.92, p for trend 0.010; mortality: HR for the highest quartile, 0.28; 95%CI, 0.10–0.76, p for trend 0.029) and mortality in colorectal cancer (HR for the highest quartile, 0.49; 95%CI, 0.26–0.90, p for trend 0.029). We also observed an increased risk of incidence and mortality in liver cancer, although no significant linear trend was observed. We found no association between neighborhood deprivation index and stomach, pancreas, lung or breast cancer. Further, no remarkable association was observed between neighborhood deprivation index and cancer survival in women.

We examined the association between neighborhood deprivation index and cancer incidence, death and survival among Cohort I after adding education as a confounder (junior high school, high school and college or higher) to the multivariate model. However, the results of these additional analyses did not substantially differ from our original analyses.

## Discussion

In this study, we found that the neighborhood deprivation index had no substantial overall association with the risk of incidence, death due to, or survival from major cancer in Japanese men and women. However, we did find positive or inverse associations for some specific sites. Among these, the risk of colorectal cancer incidence was lower in men and women with a higher neighborhood deprivation index than in those in lower index categories. Further, the risk of liver cancer incidence and mortality was increased in women with higher neighborhood deprivation. There were no significant differences in cancer survival. Results of additional analysis using Cohort I only with adjustment for education were similar to those of the original analyses.

There are several possible explanations for these findings. First, concerning the lower risk of colorectal cancer incidence and mortality in those living in more deprived areas, a national colorectal cancer screening program for middle and older aged people in Japan started in 1992, the same time as the start point of this study [23]. Thus, the effect of a delay in screening program may not fully explain these inverse associations. Rather, they might be explained by differences in lifestyle between deprived areas and less deprived areas. One risk factor for colorectal cancer is the adoption of a westernized lifestyle [24], and participants in less deprived, more westernized areas are thus at greater risk of this cancer than those in deprived areas.

Second, the strong association of infection with hepatitis B virus (HBV) and hepatitis C virus (HCV) with social class [25] suggests that higher infection rates in deprived areas might lead to a high incidence and mortality of liver cancer. Interestingly, however, we found an association in women only, and not in men. The risk of liver cancer is increased in HBV- or HCV-infected patients with heavy alcohol intake [26], and we speculate that these strong underlying risk factors mask the effect of neighborhood deprivation index on liver cancer through HBV or HCV infection in men. In contrast, a large proportion of women in this cohort did not drink alcohol, and we were therefore able to detect this putative effect in women.

Third, there is no evidence to suggest the presence of gaps in cancer treatment by neighborhood deprivation in Japan. Access to cancer treatment after diagnosis is considered to be uniform for all people in Japan thanks to the universal health coverage system [9]. This may also be applied to cancer, where most patients, regardless of socioeconomic status, are transferred to large hospitals after diagnosis and are considered to receive almost equal quality of cancer treatment. This may explain why we found no significant differences in cancer survival by neighborhood deprivation index.

Strengths of our study are its prospective design and large sample size, which yielded good statistical power to detect the effects of neighborhood deprivation. The areas included in the present study were derived from nine different areas across Japan, both north and south, and included cities, towns and villages. This caused wide variation in the distribution of deprivation-related census variables, such as household structure and occupational distribution. Our analysis also covered a wide range of cancer outcomes, which allowed the simultaneous analysis of cancer incidence, mortality and survival.

There are several limitations to our study. First, we included only middle aged Japanese at the baseline in this study. Likewise, we did not include data from metropolitan areas due to an incidental lack of cancer incidence data or insufficient deprivation index data, and our findings might therefore not be applicable to populations in these areas. Urban/rural settings may have distinct environmental and lifestyle backgrounds, with differential effects on the association between neighborhood deprivation and outcomes. A previous study in Osaka, the second largest prefecture in Japan, found poor survival in cervical and endometrial cancer among deprived women. Osaka prefecture has the highest number of households receiving welfare assistance in Japan, and the poor survival might have been due to the greater variation in neighborhood deprivation in Osaka than in the areas included in our study.

Second, we did not consider changing the neighborhood deprivation status of those study participants who moved within the study area. In our population, however, we confirmed that only 2% of the study participants fell into this category, and the failure to consider movement within the study area might not have substantially influenced the results.

Third, we did not include individual educational level as a confounding factor in our statistical model, because information on this factor was obtained from Cohort I participants only (44.5% of total participants), which would significantly reduce statistical power. However, additional sensitivity analysis which included educational level as a confounding factor using Cohort I showed similar results. Among participants who did have educational information, nearly 50% graduated from junior high school only, consistent with the generation born around World War II, when a different education system was in place. Moreover, educational level in these participants was not associated with neighborhood deprivation index or cancer outcome.

Fourth, the neighborhood deprivation index used in the present study may have been affected by a methodological limitation: the JDI was initiated based on a commonly recognized areal deprivation index in England, but the presence of similar patterns in other contexts such as Japan is unclear. A previous study identified an association between poor gastric cancer survival and the job status of unemployed and manual labor [10]. Our study covered the same data source as this study, and our additional analyses using occupation as deprivation showed similar results. On this basis, the neighborhood deprivation index appears to be an unsuitable predictor of cancer survival in Japan.

## Conclusion

The Japanese neighborhood deprivation index has no substantial association with total cancer incidence, deaths or survival in Japanese men and women. Although neighborhood deprivation index is used in many studies in European countries and shows positive associations with cancer outcome, it appears that individual deprivation such as occupation is more sensitive in detecting the association between deprivation and cancer than JDI in Japan.
